# Reproduction of forearm rotation dynamic using intensity-based biplane 2D–3D registration matching method

**DOI:** 10.1038/s41598-024-55956-z

**Published:** 2024-03-06

**Authors:** Ryoya Shiode, Satoshi Miyamura, Arisa Kazui, Natsuki Yamamoto, Tasuku Miyake, Toru Iwahashi, Hiroyuki Tanaka, Yoshito Otake, Yoshinobu Sato, Tsuyoshi Murase, Shingo Abe, Seiji Okada, Kunihiro Oka

**Affiliations:** 1 Department of Orthopaedic Surgery, Osaka University Graduate School of Medicine, 2-2 Yamadaoka, Suita, Osaka 565-0871 Japan; 2 Division of Information Science, Nara Institute of Science and Technology, 8916-5 Takayama, Ikoma, Nara 630-0192 Japan; 3 Department of Orthopaedic Surgery, Bell Land General Hospital, 500-3 Higashiyama, Naka-ku, Sakai, Osaka 599-8247 Japan; 4 Department of Orthopaedic Surgery, Toyonaka City Hospital, 4-14-1 Shibahara, Toyonaka, Osaka 560-8565 Japan

**Keywords:** Medical research, Disability, Mechanical engineering

## Abstract

This study aimed to reproduce and analyse the in vivo dynamic rotational motion of the forearm and to clarify forearm motion involvement and the anatomical function of the interosseous membrane (IOM). The dynamic forearm rotational motion of the radius and ulna was analysed in vivo using a novel image-matching method based on fluoroscopic and computed tomography images for intensity-based biplane two-dimensional–three-dimensional registration. Twenty upper limbs from 10 healthy volunteers were included in this study. The mean range of forearm rotation was 150 ± 26° for dominant hands and 151 ± 18° for non-dominant hands, with no significant difference observed between the two. The radius was most proximal to the maximum pronation relative to the ulna, moved distally toward 60% of the rotation range from maximum pronation, and again proximally toward supination. The mean axial translation of the radius relative to the ulna during forearm rotation was 1.8 ± 0.8 and 1.8 ± 0.9 mm for dominant and non-dominant hands, respectively. The lengths of the IOM components, excluding the central band (CB), changed rotation. The transverse CB length was maximal at approximately 50% of the rotation range from maximum pronation. Summarily, this study describes a detailed method for evaluating in vivo dynamic forearm motion and provides valuable insights into forearm kinematics and IOM function.

## Introduction

Forearm rotation is a very important function in daily life^1^, with the normal range of motion generally considered to be 180°^2,3^. The three-dimensional (3D) complex morphological structure of the radius and ulna allows for a large range of forearm motion. This rotational motion is primarily a rotation of the radius relative to the ulna, accompanied by a minor translation of the radius in the axial, coronal, and sagittal planes^4^. The interosseous membrane (IOM) of the forearm constitutes a stable fibrous tissue that facilitates the connection between the radius and ulna^3^. Alongside the proximal (PRUJ) and distal (DRUJ) radioulnar joints, it assumes a pivotal role in governing forearm rotation and conducting load^4–8^. The IOM of the forearm forms a complex structure comprising the proximal oblique cord (POC), dorsal oblique accessory cord (DOA), central band (CB), distal accessory band (DAB), and distal oblique bundle (DOB)^9,10^. While there have been numerous reports on CB dynamics^9,11,12^ of the IOM, the roles of other components have not been elucidated. In vivo studies of forearm rotation dynamics have been conducted using two-dimensional (2D) data from plain radiograph^13,14^, computed tomography (CT)^15,16^, and magnetic resonance imaging (MRI)^17^. Recently, research on 3D dynamic analysis using 3D computer bone models generated from CT images in multiple positions has been conducted^18–21^. However, these analyses were based on static image data and did not constitute true 3D dynamic analyses.

A method known as “2D–3D registration” enables true 3D dynamic analysis by combining static 3D images, such as CT images, with dynamic 2D images, such as fluoroscopic images. This method was first developed for the dynamic analysis of artificial joints^22^ and has been used for knee^23^ and shoulder^24^ joint dynamic analyses in vivo. While dynamic analysis of the forearm has been performed using single-plane serial fluoroscopic images^25–27^, only a few reports exist using biplane imaging, despite their superior accuracy in registration matching^28,29^. Therefore, we developed a unique intensity-based biplane 2D–3D registration matching method that uses density gradient information^30,31^ between fluoroscopic and CT images to perform dynamic analysis^32^ at 12.5 fps, resulting in a truly dynamic analysis method compared with the techniques used in previous reports^18–21,25^.

In this study, we aimed to reproduce and analyse the dynamic forearm rotation including axial translation of the radius and length of IOM of the forearm using the intensity biplane 2D–3D registration matching method.

## Results

### Measurement of range of motion

Our analysis of 20 upper limbs from 10 healthy volunteers revealed that the mean range of forearm rotation was 68.8 ± 12.5° for the dominant hand and 70.8 ± 17.5° for the non-dominant hand in pronation, 82.4 ± 14.2° for the dominant hand and 79.3 ± 13.2° for the non-dominant hand in supination, and 150 ± 26° for the dominant hand and 151 ± 18° for the non-dominant hand in total. No significant differences were found between dominant and non-dominant hands (*p = *0.78 in pronation, *p = *0.63 in supination, and *p = *0.57 in total). The neutral position was 46% for the dominant hand and 47% for the non-dominant hand from maximum pronation.

### Measurement of axial translation of the radius relative to the axis of rotation

**Figure 1:**
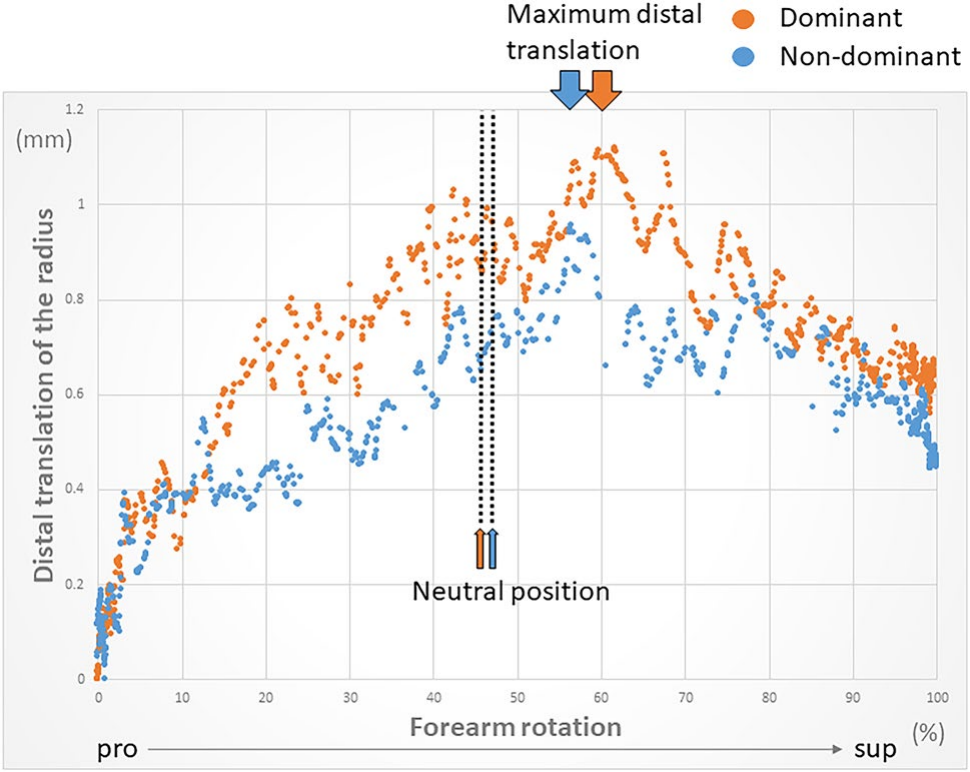
The kinematics of forearm rotation of 20 upper extremities of 10 participants were evaluated by averaging values in dominant and non-dominant hands. Forearm rotation was evaluated as 100% of the full range of motion and as a percentage of the maximal rotational position. Distal translation of the radius relative to the forearm axis of rotation is expressed. The radius moved most distally near the neutral position.

The mean range of axial translation of the radius was 1.8 ± 0.8 mm for the dominant hand and 1.8 ± 0.9 mm for the non-dominant hand during forearm rotation. No significant difference was found between dominant and non-dominant hands (*p = *0.89). The radius was most proximal in maximum pronation, moved distally toward the neutral position, and after reaching the most distal position, moved proximally again toward supination (Fig. 1). Forearm rotation wherein the radius was most distal was 62% for the dominant hand and 56% for the non-dominant hand from maximum pronation, while the radius was 0.85 ± 0.96 mm for the dominant hand and 0.99 ± 0.78 mm for the non-dominant hand, which is more proximal in the maximum pronation position than in the maximum supination position. No significant difference was found between dominant and non-dominant hands (*p = *0.31).

### Measurement of lengths of the ligaments stabilizing the IOM

**Figure 2:**
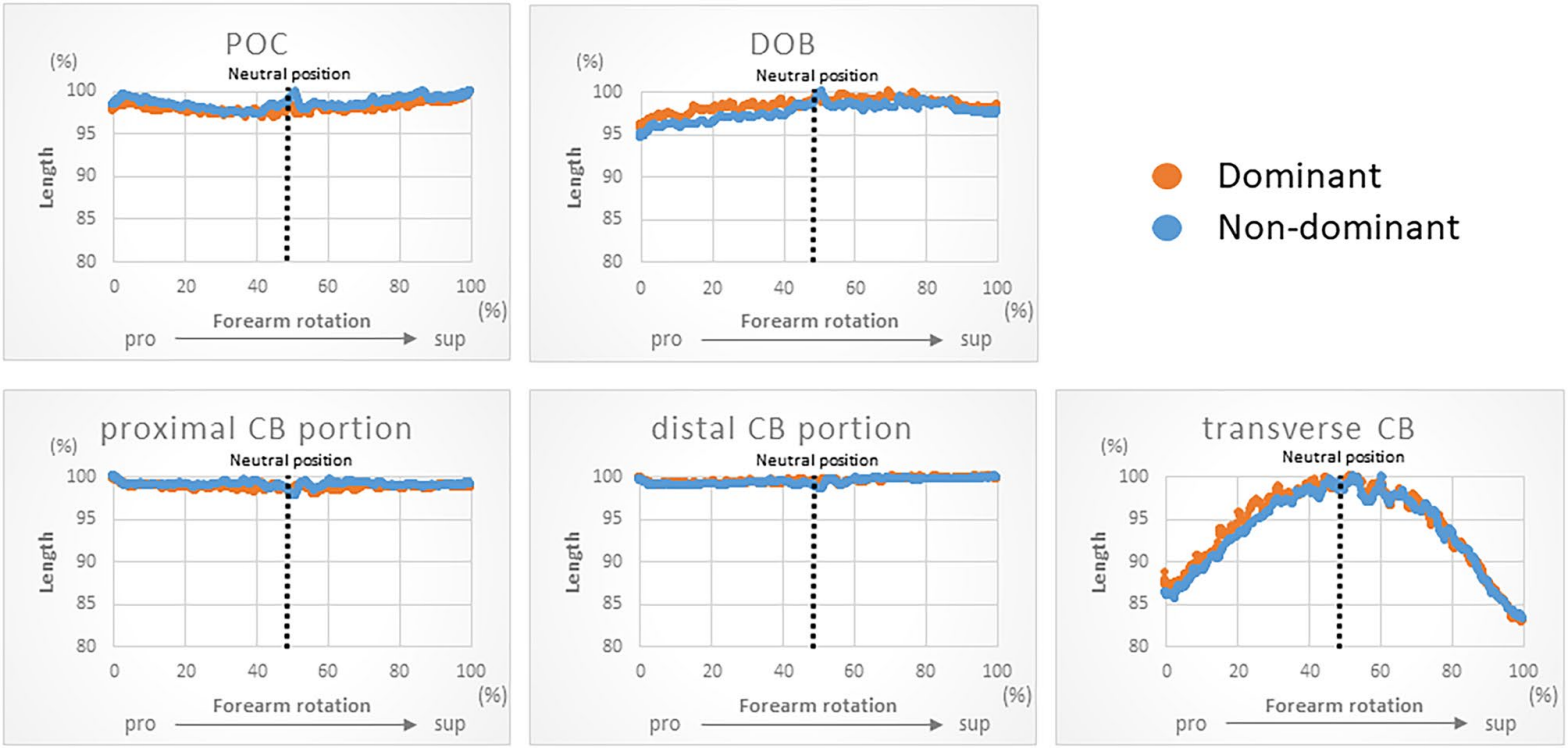
The kinematics of each component of the IOM were evaluated by averaging values in dominant and non-dominant hands. Forearm rotation was evaluated as 100% of the full range of motion and as a percentage of the maximal rotational position. The transverse of the CB, which is the distance between the distal CB attachment of the radius and proximal CB attachment of the ulna, was measured to determine the interosseous distance.

The lengths of the POC, proximal CB portion, distal CB portion, and DOB varied by 3 ± 0.6%, 2 ± 0.4%, 1 ± 0.3%, and 4 ± 0.9% for the dominant hand and 3 ± 0.6%, 2 ± 0.3%, 2 ± 0.4%, and 5 ± 1% for the non-dominant hand of the total length, respectively (Fig. 2). POC was longest in the pronation and supination positions and shortest in the neutral position, CB showed little change during rotation, and DOB was shortest in the pronation position and longest from the neutral to the supination position. The transverse CB lengths varied by 17 ± 5% of the total length for both the dominant and non-dominant hands. Forearm rotation wherein the maximal transverse CB was 52% from maximum pronation for the dominant hand and 53% for the non-dominant hand.

## Discussion

In this study, the axial motion of the radius with respect to the rotation axis was reproduced from biplane fluoroscopic images during dynamic forearm rotation in healthy individuals, along with the estimated IOM length in the forearm, analysed using a unique intensity-based biplane 2D–3D registration matching method^32^. Our previous report showed the high accuracy of this method for forearm measurements, with rotational mean absolute errors (MAEs) ± standard deviation (SD) of 0.31 ± 0.35° and 0.32 ± 0.33° and translational MAE ± SD of 0.43 ± 0.35 mm and 0.29 ± 0.25 mm in the radius and ulna, respectively^32^. To our knowledge, this is the first report to reproduce and analyse forearm rotational dynamics in vivo using serial biplane fluoroscopic images.

In this study, the mean range of forearm rotation measured was 150 ± 26° for dominant hands and 151 ± 18° for non-dominant hands, which is comparable to results from previous reports^21,25^. Since the clinical range of motion for pronation and supination encompasses the carpal^33^, forearm, and humeroulnar^34^ joints, the range of motion of the forearm alone appears narrower than the clinical range of motion.

The mean range of axial translation of the radius along the axis of rotation was 1.8 ± 0.8 mm for dominant hands and 1.8 ± 0.9 mm for non-dominant hands. These values were greater than the translational MAE of this 2D–3D registration method^32^. Previous studies have reported a mean range of axial translation of the radius of 1.9–2.3 mm during forearm rotation using 3D evaluation in multiple positions^19,21^, which corresponds to the amount of translation. Although the radius is generally considered to move distally from pronation to supination^13,35^, some reports have claimed that this bone moves most distally in the neutral positon^21^; however, this has yet to be fully established. This study showed that the most distal movement occurred at 62% (dominant hands) and 56% (non-dominant hands) of the total range of motion from the maximum pronation position. Since the neutral position was 46% and 47% for the dominant and non-dominant hands, respectively, from maximum pronation, we found that the most distal movement occurred at the neutral to slightly supinated position. Our study generally supports a 2D–3D registration study using static biplane radiographs in multiple positions^21^, but the difference is that the present study analysed true dynamic dynamics, which revealed that the most distal translation occurs at neutral to slightly supinated positions. The difference from previous studies^13,35^, in which the radius moved distally towards supination during forearm rotation, may be due to the use of 2D evaluation only or the small number of positions evaluated.

**Figure 3:**
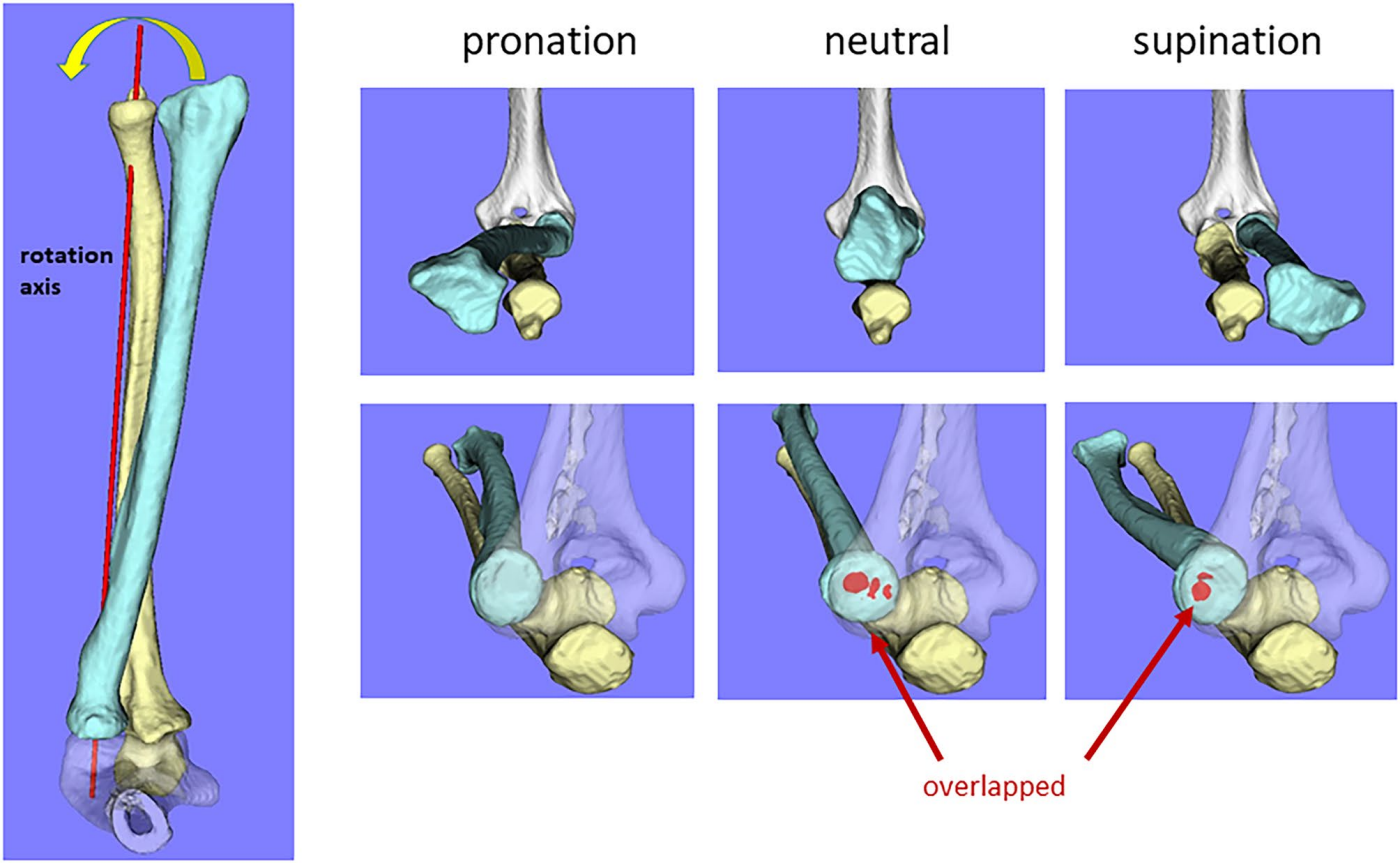
Analysis of the mechanism of distal translation of the radius performed using computer simulation. Rotation of the radius from pronation to supination around the rotation axis using only the rotational component of the actual dynamics. In neutral and supination positions, the radius was observed to overlap the humerus at the humeroradial joint, but in actual mechanics, which includes a translational component, the radius was expected to translate distally to maintain congruency at the humeroradial joint.

Forearm rotation from pronation to neutral causes the radius to move distally and from neutral to supination slightly proximally, which is thought to depend on the congruency in the humeroradial joint^16,36^. Based on computer simulation, the radius rotates from pronation to neutral and then to supination along the rotational axis only with the rotational component and without translation (Fig. 3). In neutral and supination positions, the radial head was observed to overlap the capitellum at the humeroradial joint. Greater overlap was observed in the neutral than in the supination. However, in actual in vivo motion, the radius was expected to translate distally to maintain congruency at the humeroradial joint. The distal translation of the radius significantly impacts the clinical assessment of the ulnar variance. Ulnar variance is associated with wrist disorders, such as Kienböck disease^37^ and ulnar impaction syndrome^38^. Our results suggest that radiographic evaluation of the wrist in the pronated position is necessary, particularly when ulnar impaction syndrome is suspected, as ulnar variance can be underestimated by a mean of 1.8 mm when anteroposterior radiographs of the wrist are performed in the neutral position.

The IOM is a vital structure that provides longitudinal stability to the forearm during rotational movements, yet its dynamics in vivo remain unclear. In this study, we created a virtual IOM and analysed its in vivo dynamics. The CB showed minimal change in length during rotation, consistent with previous reports^39,40^, but smaller than that of the other components, suggesting that CB is a main stabilizer of IOM during forearm rotation^39^. The length change in the POC (3%) was lower than the values reported in the previous study (14%)^40^. The DOB (4–5%) ranges were similar to those reported previously (4%)^39,40^. The difference between the findings of the present and previous studies may be attributed to the measurements under dynamic and static conditions. Alternatively, in previous studies, the images were acquired with the elbow in full extension, whereas in this study, the images were acquired with the elbow in 90° flexion, which can alter forearm mechanics^41^. The biceps brachii, one of the extrinsic muscles of the forearm, is particularly influenced by elbow flexion, affecting the proximal IOM components, POC, while the distal components, DOB, are less affected. The narrow range of variation suggests that POC and DOB are the stabilizers of PRUJ and DRUJ, respectively. Since DOB begins at the distal end of the ulna and continues with the triangular fibrocartilage complex to the sigmoid tuberosity of the radius, DOB may be a stabilizing factor for the DRUJ^9,42,43^. A report has indicated that POC may play some role in the stability of elbow and wrist rotation^44^, and the results of this study support that POC contributes to PRUJ stability.

Transverse CB is used as a measure of interosseous distance^18^. In this study, the range of change was 17%, with the most laxity at the supination position and the most tension at the neutral position, similar to previous reports^18,45,46^. Although IOM strains have been reported to be maximal at the neutral position, the proximal and distal CB portions do not show any change in length during forearm rotation. As the transverse CB shows the most tension at the neutral position, it was a useful quantitative indicator of the strain of the CB, which is the toughest component of the IOM. Although the forearm is often immobilized in the neutral position for forearm and wrist trauma, the results of this study support that immobilization of the forearm in the neutral position is recommended to prevent contracture of the IOM.

This study had a few limitations. First, the models under consideration in this study are limited to bone alone. Nevertheless, dynamic bone motion encompasses the influence of soft tissue effects, and our method aligns more closely with genuine dynamics than the static method. Second, in this study, the amount of translation of the radius relative to the ulna was calculated as the amount of translation relative to the forearm rotational axis. It is possible that the results, which is movement on the Z-axis, is a slight overestimation since the UV is measured based on the longitudinal axis of radius. Third, this study is based solely on the assumption that all components are present in all individuals. Although the IOM was modelled from the attachment in previous reports, some components of the IOM are not present in all individuals.

Summarily, intensity-based biplane 2D–3D registration methods were applied to analyse the dynamic forearm rotation, including axial translation of the radius, and determine the anatomical function of the IOM. The radius moved distally from pronation to supination, reaching the most distal position at approximately 60% of the rotation range. The lengths of the IOM components other than the CB were characterized as changing during rotation. The transverse of the CB was maximal at the neutral position of the rotation range.

## Methods

### Participants

Ten healthy male volunteers (mean age, 36.9 years; range, 32–54 years) without a history of bilateral upper extremity trauma or disease were recruited between 2020 and 2021. Twenty upper extremities from the 10 participants were included in the analysis.

### CT images

CT scans of volunteer bilateral forearms were performed in the supination position with a low-dose radiation protocol^47^ (slice intervals: 1.25 mm; tube voltage: 120 kV; tube current: 10 mA; and helical pitch: 0.562:1). Segmentation and reconstruction of the bone surface models of the humerus, radius, and ulna were performed using the commercial software Bone Viewer (Teijin Nakashima Medical Co., Okayama, Japan).

### Biplane fluoroscopic images

**Figure 4:**
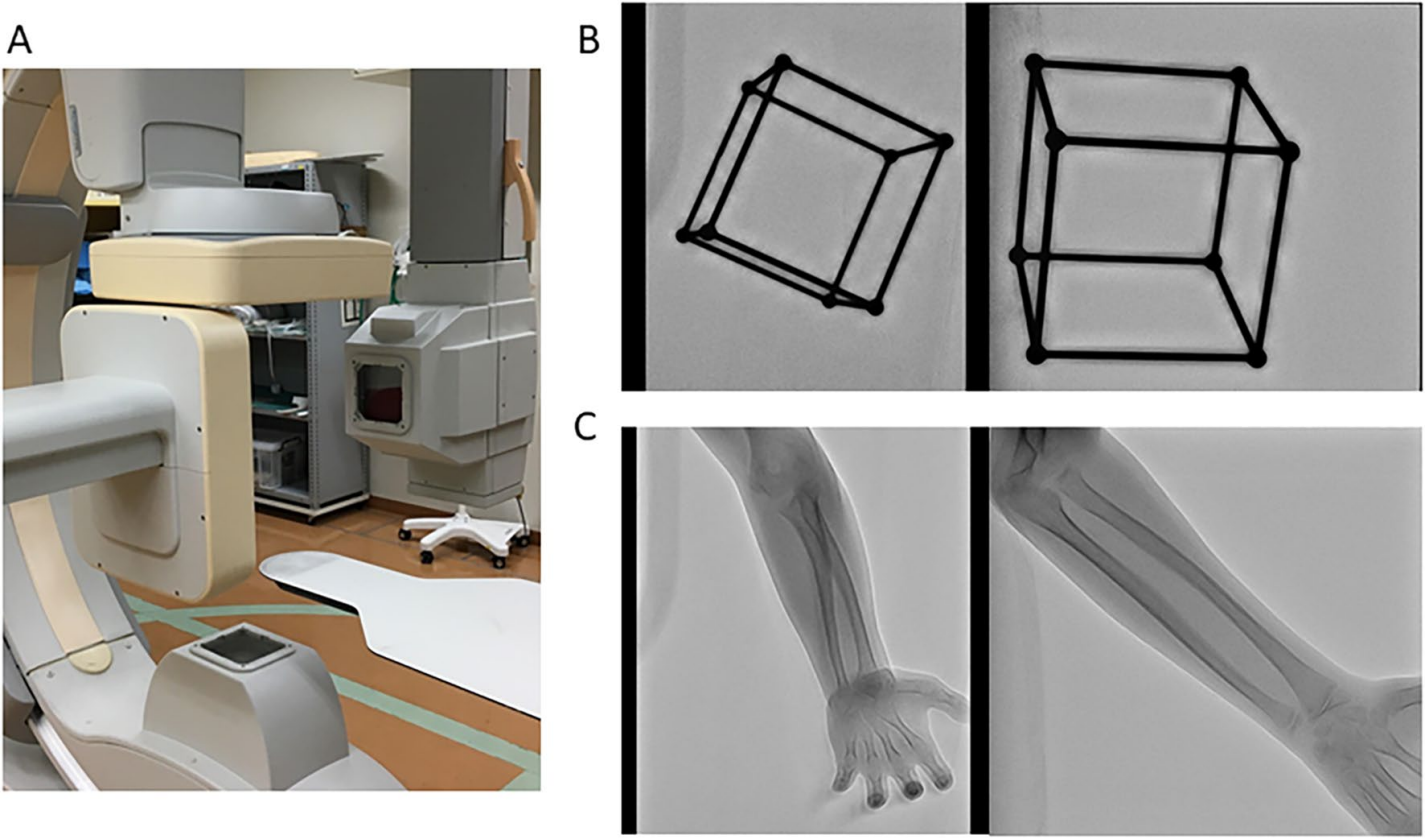
Method of acquisition of continuous fluoroscopic biplane images. (**A**) The two sources and detectors are placed in a 90º position. (**B**) The calibration frame is taken, and calibrations of the images in the two directions are performed. (**C**) Forearm rotation motion is performed with the 90º flexion position of the elbow joint.

Biplane fluoroscopic images were obtained using a biplane C-arm (Allura Clarity FD20/20; Philips Healthcare, Amsterdam, The Netherlands) (Fig. 4A). The X-ray source and detectors, anterior–posterior and lateral, were positioned perpendicularly. The imaging conditions were as follows: frames rate: 12.5 fps; field-of-view: 378 × 378 mm with an image resolution of 1024 × 1024 pixels (pixel size: 0.37 mm) in anterior–posterior view and 292 × 292 mm with an image resolution of 1024 × 1024 pixels (pixel size: 0.29 mm) in lateral view; output power was “auto” because tube voltage and tube current were not manually adjustable in this equipment. To synchronise the images in the two directions, the C-arm generated X-rays at 12.5 fps every 40 ms alternately in the anteroposterior and lateral directions. Before commencing the imaging procedure, biplane images of the calibration box designed to be 11 cm between the corners were acquired to calculate the relative positions of the two X-ray sources and the distance between the X-ray sources and detector^48^, and a global coordinate system was set up on the biplane equipment (Fig. 4B). After setting the conditions for biplane fluoroscopic images, each volunteer rotated his forearm from full supination toward full pronation during imaging and then back toward full supination, with the elbow flexed at approximately 90° and within 10 s (Fig. 4C). Regarding the size of the detector, the entire length of the forearm could be included in the imaging range; however, only a portion of the distal end of the humerus could be included. Because of the difficulty in obtaining an accurate registration, we did not include the humerus in registration in this study.

### Intensity-based 2D–3D matching method (2D–3D method)

**Figure 5:**
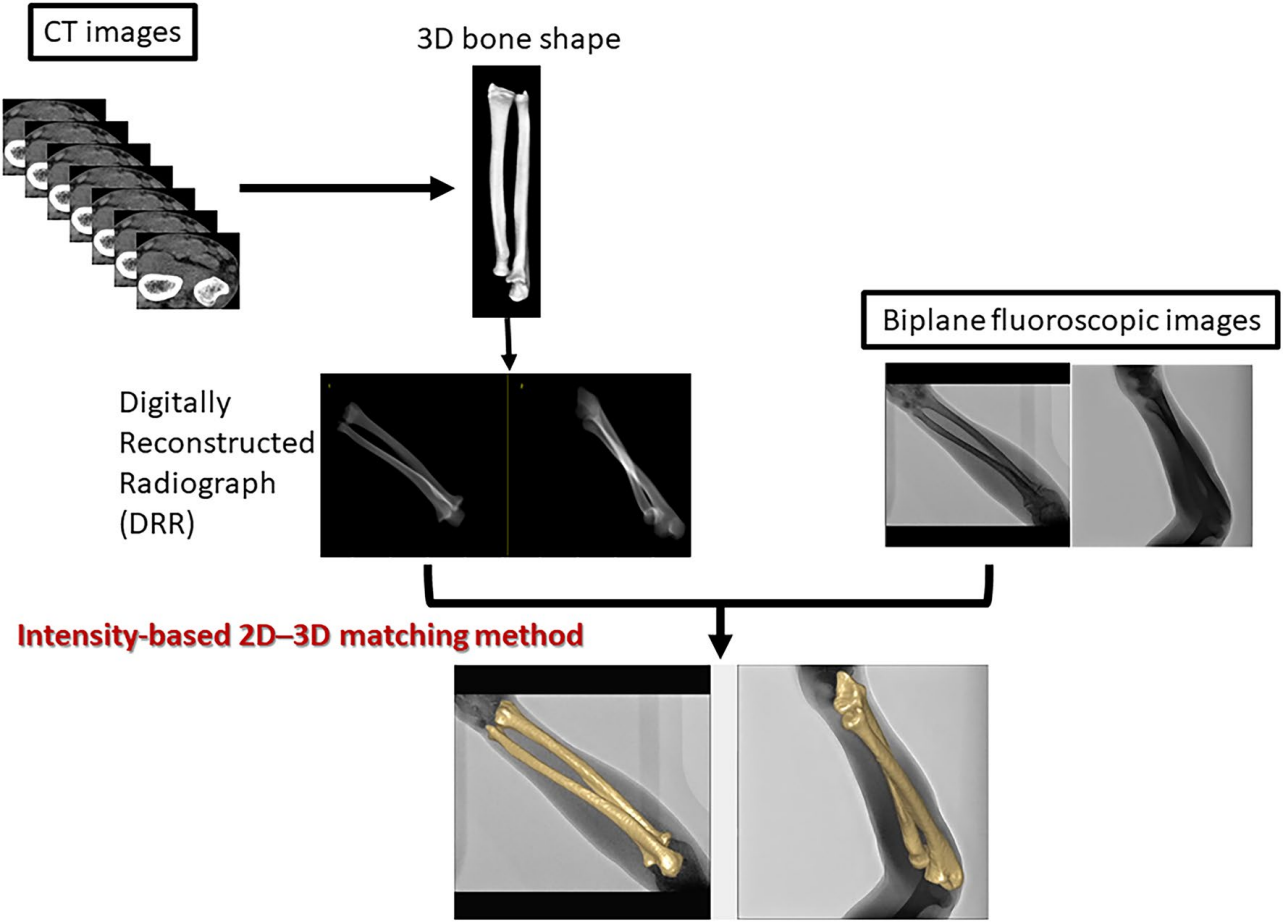
Schematic diagram of intensity-based 2D–3D matching method, in which DRR images are generated from a CT image, and the 3D bone shape obtained from CT is superimposed on the biplane fluoroscopic images based on the density gradient information in the DRR images and that in the biplane fluoroscopic images.

The intensity-based 2D–3D matching method is depicted in Fig. 5^32^. Biplane fluoroscopic images were obtained as 2D dynamic information, and CT images were obtained as 3D static information. In the intensity-based 2D–3D matching method, the 3D position of the target bone during forearm rotation was calculated using the pixel intensity of the image. In our method, the similarity between the density information in the fluoroscopic image and the digitally reconstructed radiograph (DRR) generated from the CT was automatically matched by evolutionary optimisation using MATLAB (The MathWorks Inc., Natick, MA, USA). The bone position in the first frame was set by manual matching, and for subsequent frames, bone matching was automatically performed using the calculated value of the previous frame as the initial value.

### Coordinate system

**Figure 6:**
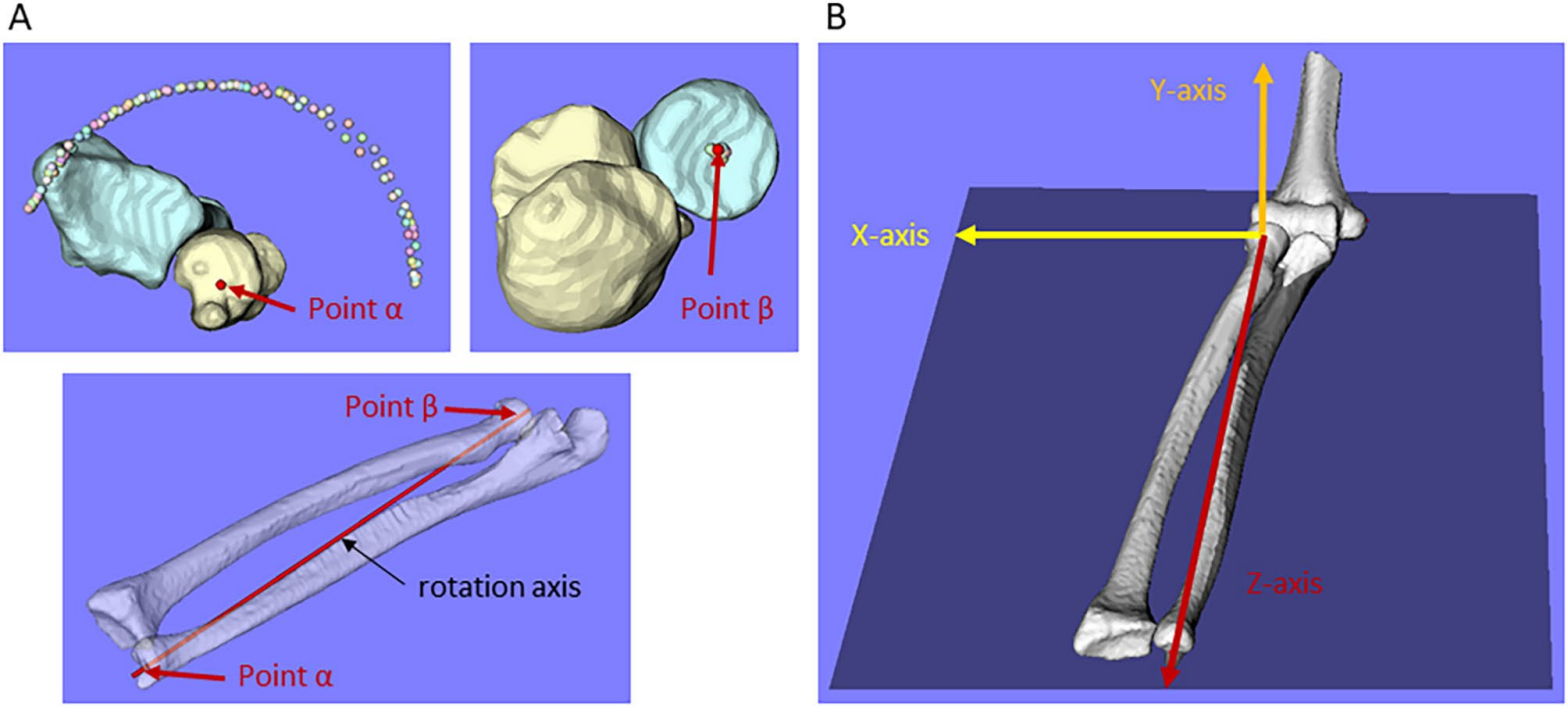
(**A**) The line connecting points α and β is defined as the axis of rotation (Z-axis). (**B**) The X-axis is defined as a line perpendicular to the Z-axis and parallel to a line passing through the top of the humeral medial and lateral epicondyles and passing through the origin; a line perpendicular to both the X- and Z-axes is used as the Y-axis.

The tip of the radial styloid process and centre of the radial head were specified manually, and the centre of the approximate circle fitted to the trajectory of the tip of the radial styloid process (point α) and the approximate circle fitted to the trajectory of the centre of the radial head (point β) were calculated by 2D–3D matching (Fig. 6A). Point β was used as the origin, and the line connecting the two points (points α and β) was defined as the rotation axis, which was used as the Z-axis. The X-axis was defined as a line perpendicular to the Z-axis and parallel to a line passing through the top of the humeral medial and lateral epicondyles modelled at the CT imaging position and passing through the origin. The Y-axis was defined as a line perpendicular to both the X- and Z-axes (Fig. 6B). The X-axis was defined as positive in the lateral direction and negative in the medial direction, the Y-axis was defined as positive in the palmar direction and negative in the dorsal direction in the supinated position, and the Z-axis was defined as positive in the distal direction and negative in the proximal direction. All evaluations were performed based on the motion of the radius relative to the ulna.

### Measurement of range of motion

**Figure 7:**
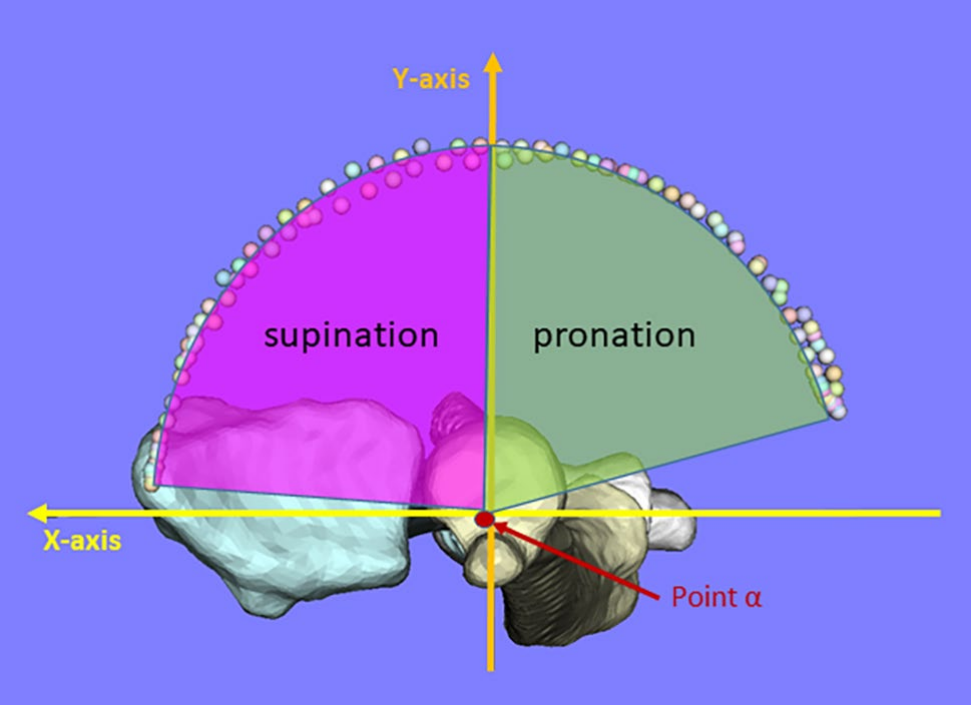
Definition of forearm rotational range of motion; the angle is calculated by projecting the trajectory of the radial styloid process in the XY plane, and each is calculated for pronation and supination motions.

The forearm rotational range of motion in the 3-D bone model was measured by modifying the method previously reported by Crisco et al.^49^ The angle between the line connecting the origin and radial styloid process and the X-axis was calculated in the XY plane viewed distal to the Z-axis (Fig. 7). The neutral position was defined as when the angle between the line connecting the origin and the radial styloid process and the Y axis is 0 degrees.

### Measurement of axial translation of the radius

The movement of the whole radius along the Z-axis was measured as the distal/proximal translation of the radius during forearm rotation. When measuring this axial translation of the radius, forearm rotation was expressed as the percentage of maximum pronation (0%) to maximum supination (100%).

### Measurement of the lengths of the IOM

**Figure 8:**
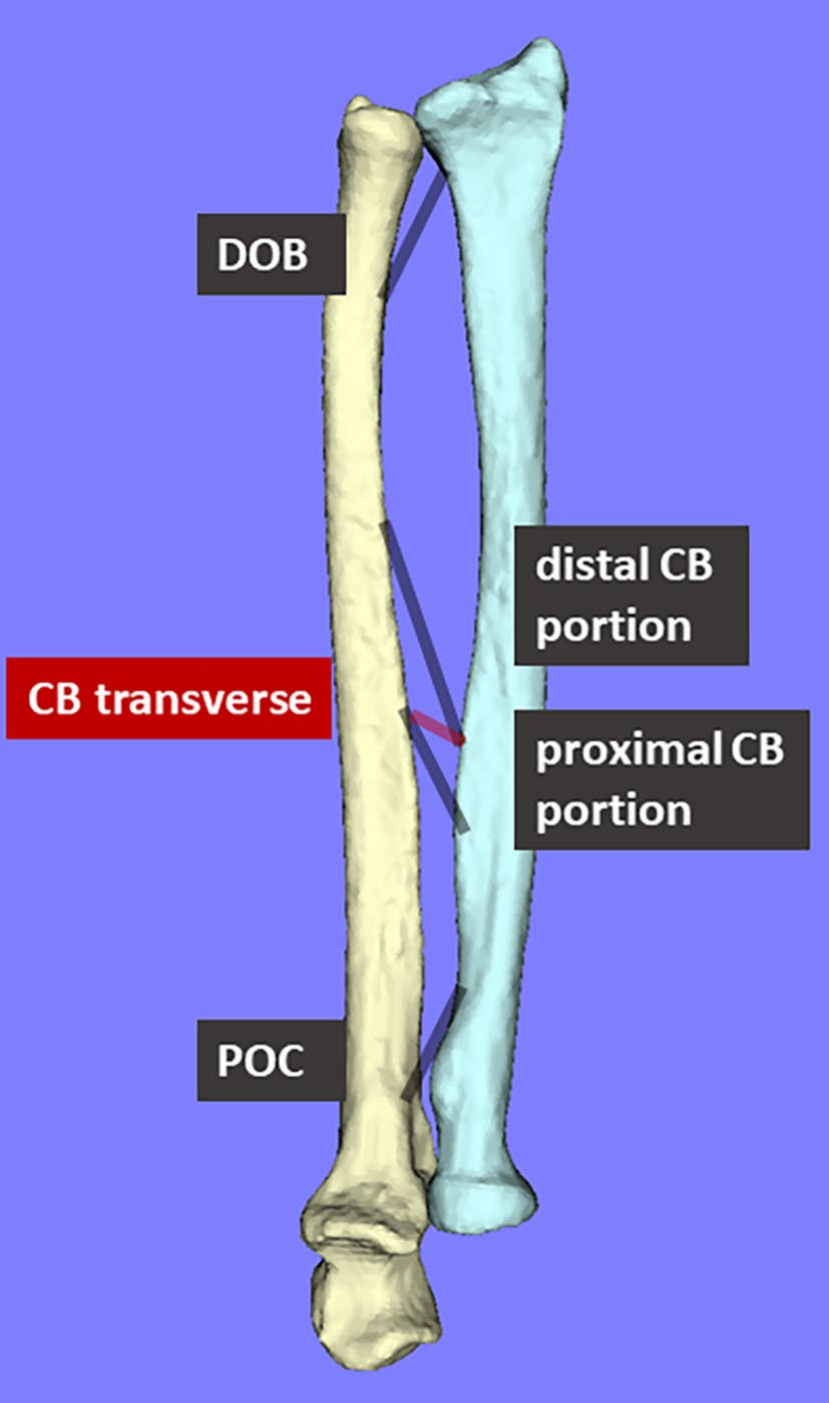
Five components (POC, CB, and DOB) were defined, as well as the transverse CB and the distance between the distal CB attachment of the radius and proximal CB attachment of the ulna.

In this study, the IOM was divided into three components: POC, CB, and DOB. Since DOA and DAB are tissues near the CB, they were excluded from this analysis. The CB was further divided into proximal and distal portions. Each component was manually modelled on a computer model according to anatomical landmarks from previous reports^43^ (Fig. 8). A point 80% of the total length from the distal end of the ulna was identified as the ulnar origin of the POC, and a point 79% of the total length from the distal end of the radius was identified as the radial insertion of the POC. A point 64% of the total length from the distal end of the radius was identified as the radial origin of the proximal portion of CB, and a point 44% of the total length from the distal end of the ulna was identified as the ulnar insertion of the proximal portion of CB. A point 53% of the total length from the distal end of the radius was identified as the radial origin of the distal portion of CB, and a point 29% of the total length from the distal end of the ulna as the ulnar insertion of the distal portion of CB. A point 15% of the total length from the distal end of the ulna was identified as the ulnar origin of the DOB, and a point 9.9% of the total length from the distal end of the radius as the radial insertion of the DOB. The shortest paths of each component during forearm rotation were modelled and measured. For the analysis, the dynamics of all cases were averaged, and the range of change for each component was measured with a maximum length of 100%. Forearm rotation when each component was the longest and shortest were expressed as percentages of the maximum pronation at 0% when the full range of motion was set at 100%. The transverse of CB was measured to evaluate the interosseous distance and was set according to a previous report^18^.

### Statistical analysis

All statistical analyses were performed using JMP Pro 14 software (SAS, Cary, NC, USA). We used nonparametric statistical analyses to validate values between dominant and non-dominant hands. A *p*-value < 0.05 was considered statistically significant.

### Ethical approval and consent to participate

This study was approved by the Institutional Review Board of Osaka University Hospital (approval no. 15521) and was conducted in accordance with the Declaration of Helsinki. Written informed consent was obtained from all participants.

## Data Availability

The datasets generated and/or analysed during the current study are available from the corresponding author upon reasonable request.
